# Graph Theory of Tower Tasks

**DOI:** 10.3233/BEN-2012-0345

**Published:** 2011-12-29

**Authors:** Andreas M. Hinz

**Affiliations:** ^1^Mathematisches InstitutLudwig-Maximilians-Universität MünchenTheresienstraße 3980333 MünchenGermany; ^2^Fakultät für Mathematik und InformatikFern Universität in HagenLützowstraße 125, 58095 HagenGermany

**Keywords:** Tower of Hanoi, Tower of London, mathematical model, graph

## Abstract

The appropriate mathematical model for the problem space of tower transformation tasks is the state graph representing positions of discs or balls and their moves. Graph theoretical quantities like distance, eccentricities or degrees of vertices and symmetries of graphs support the choice of problems, the selection of tasks and the analysis of performance of subjects whose solution paths can be projected onto the graph. The mathematical model is also at the base of a computerized test tool to administer various types of tower tasks.

## 1. Puzzles and graphs

Informally, a *graph* consists of a set of labelled dots, every pair of which may or may not be joined by lines. The concept originates in the 19th century with *chemical graphs*, where dots represent atoms and lines stand for bonds between them (cf. [3, Chapter 4]). The term “graph” as used in this note must not be confused with the word employed for the graphical representation of a function. An everyday example of a graph is an underground network map, where the dots represent stations and neighboring stations on underground lines (!) are linked on the map.

Graphs have turned out to be a suitable mathematical model to analyze puzzles. Participants of the Heybeliada RGACD meeting might have posed themselves the following question. Consider the topography of İstanbul with the ancient Constantinople (C), Galata (G), Üsküdar (Ü), and the island of Heybeli (H). There are two bridges (Galata and Atatürk) between C and G, two (Bosporus and Fatih Sultan Ahmet) between G and Ü, and ferries from C, G, and Ü to H. Here is the problem: is it possible to make a round trip using every transfer connection (four bridges and three ferries) exactly once? The solution is given by looking at the graph in Fig. 1.

If such a tour were possible, you would enter any dot as many times as you leave it, so the number of lines attached to each dot must be even. However, they are all odd in our graph.

This problem is equivalent to the famous *Königsberg Bridges problem* (cf. [3, Chapter 1]), which was solved in 1736 by the Swiss mathematician Leonhard Euler (1707–1783), who thereby already used the concept of graph implicitly. (A closed trail in a graph, passing every line exactly once is called an *eulerian circuit*.) Equivalence for graphs means that they are *isomorphic*, which is to say that they have the same form (same number of dots associated in the same way by lines).

A graph was also the underlying feature of a puzzle invented by the Irish physicist and mathematician William Rowan Hamilton (1805–1865), namely the *Icosian Game*; cf. [3, Chapter 2]. It is played on a *dodecahedral graph*, i.e., the stereographic projection of the dodecahedron, one of the five Platonic bodies; see Fig. 2.

The twenty vertices of this body are labelled with the pure consonants of the English alphabet, and the task is to start in some vertex, walk along the edges of the dodecahedron to pass every vertex precisely once and finally to return to the starting point. This is called a *hamiltonian cycle* on the graph and in fact, the dodecahedral graph is *hamiltonian*, i.e., admits such a cycle. (Follow the letters in alphabetic order!)

[1] ©A.M.Hinz 2011.
*Corresponding author. E-mail: hinz@math.lmu.de.

ISSN 0953-4180/12/$27.50 © 2012 – IOS Press and the authors. All rights reserved

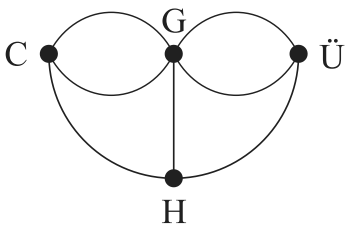

Fig. 1. Topography of İstanbul with Constantinople (C), Galata (G), Üsküdar (Ü), Heybeliada (H) and transfer connections.

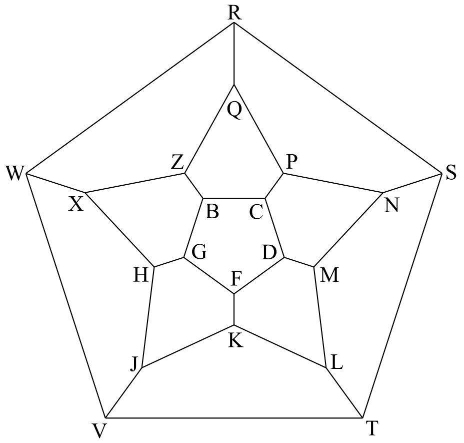

Fig. 2. Hamilton's dodecahedral graph.

## 2. Formal definition of a graph

Following the last example, it has become customary to call the dots in a graph *vertices* and the lines *edges*. Therefore, as a mathematical object, a (*simple*) *graph* $G$ is defined as a pair $(V, E)$, where $V$ is some set and $E$ is a subset of $\binom{V}{2} := \{\{x, y\}; x, y \in V, x \neq y\}$; $E$ is also called an *association* on $V$.

With this formal definition we exclude the possibility of multiple edges as present in Euler's graph, because we will not need them anymore. Two graphs $(V, E)$ and $(V', E')$ are then *isomorphic*, if there is a one-to-one correspondence between the sets $V$ and $V'$ and such that a pair of vertices in $V$ forms an edge in $E$ if and only if the corresponding pair in $V'$ forms an edge in $E'$. Details from graph theory can be found, e.g., in [32].

## 3. State graphs of solitaire games

Solitaire games are characterized by a set of admissible or reachable states of some material and rules describing moves to get from one state to another one. Tower puzzles consist of a certain number of distinguishable objects (discs of different size in the Tower of Hanoi (ToH) or balls of different color in the Tower of London (ToL)) which must be arranged on three (or more) vertical pegs, such that only the topmost disc/ball on a peg is free to move. Apart from the general rule that only one object may be transferred at a time from one peg to the other, further restrictions on the moves can be imposed like the *divine rule* for the ToH: "You must not place a larger disc on a smaller one!". The name derives from the legendary origin of the *Tower of Brahma* to which the inventor of the puzzle, the French mathematician Édouard Lucas (1842–1891), alludes. (Its 64 discs were made of gold and decorated with diamonds from the famous Golconda market in Hyderabad, the venue of the next RGACD meeting!) In the ToL, the pegs can only hold a limited number of balls, respectively.

It is therefore natural to model tower puzzles by a graph whose vertices are the regular distributions of discs/balls on the pegs and whose edges represent legal moves. In psychology literature, the term *problem space* has been used as a synonym for what we call a *state graph*. We will avoid the former expression because it contains additional details for the problem solver like, e.g., how to move the objects physically etc. (cf. [23]).

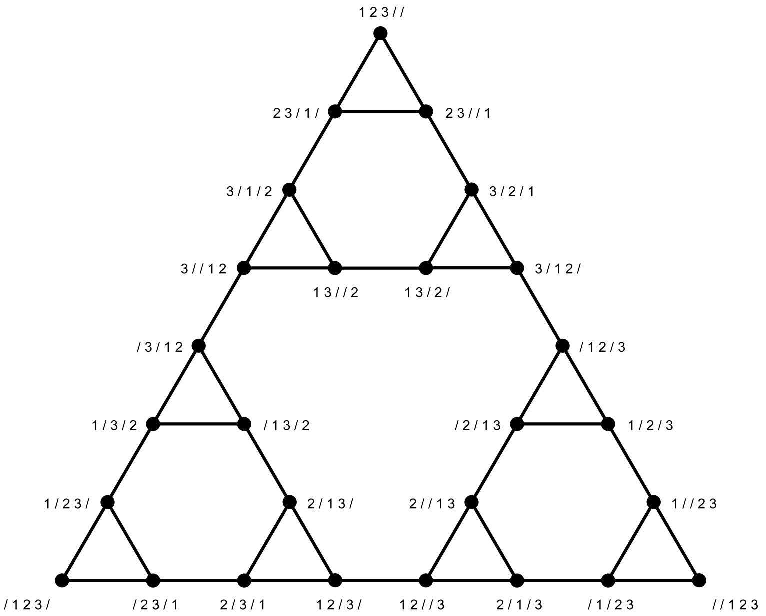

Fig. 3. Hanoi graph $H_3^3$.

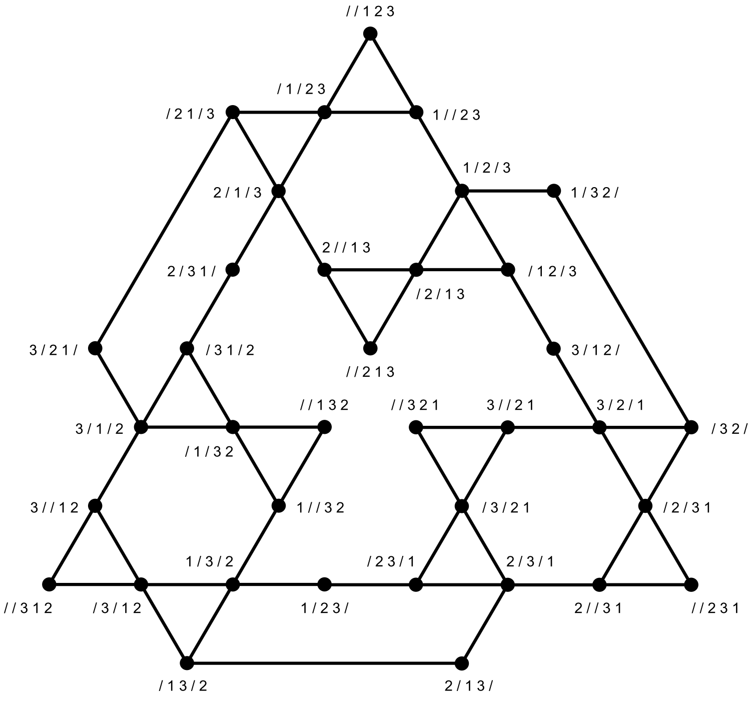

Fig. 4. London graph $L_{123}^3$.

Figures 3 and 4 show the examples of the graphs $H_3^3$ for the ToH with three discs (cf. [26]) and $L_{123}^3$ for the classical ToL (cf. [5]), respectively. (More general, *Hanoi graph* $H_p^n$ stands for the state graph of a ToH with $p \geqslant 3$ pegs and $n \in \mathbb{N}$ discs, and *London graph* $L_{h_1 \ldots h_p}^n$ represents a ToL with $n \in \mathbb{N}$ balls and $p$ pegs of heights $h_1, \ldots, h_p$, where we will assume that $1 \leqslant h_1 \leqslant \ldots \leqslant h_p \leqslant n$.)

For the labelling of the vertices we make use of the following notation for states of the respective puzzles.

The discs or balls are numbered from 1 to $n$ according to size or color. We then write down, from left to right, the objects (if any) on the first peg from top to bottom, then note the bottom of the first peg by a slash $/$, continue with the objects on the second peg and so on. Thus $12/3/$ stands for the state in $H_3^3$ with disc 1 on top of disc 2 on the first peg, disc 3 alone on the second and with the third peg empty. (Obviously, we do not need to write down a slash for the bottom of the third peg.) Similarly, $1//32$ is the state in $L_{123}^3$ where the ball colored with color 1 lies on the first (shortes) peg, the medium size peg is empty, and ball colored 3 is on top of ball colored 2 on the tall peg.

## 4. Mathematical analysis of tower graphs

Analyzing the state graphs reveals a wealth of information for the use of the puzzles in psychological tests. First, it is obvious from Figs 3 and 4 that $H_3^3$ and $L_{123}^3$ are *not* isomorphic. As a consequence, the puzzles are *not* equivalent as a test, because their problem structure is reflected in the state graphs. Note, however, that conversely isomorphy of state graphs, i.e., mathematical equivalence, does *not* imply psychological equivalence.

This is impressively demonstrated by the example of "Monsters and Globes" (MaG). Here (see [19, p. 251] for details) the presentation of the ToH puzzle is changed from pegs and discs to monsters and globes in order to produce a *problem isomorph*. In one version (MaG move), globes ($\approx$ discs, but in inverted order of size) are exchanged between monsters ($\approx$ pegs coded by size of monsters), but now the condition that only the topmost disc can be moved in the ToH for mechanical reasons has to be stated as an extra rule "only the largest globe held by a monster may be transferred". In the second version (MaG change), monsters hold one globe each whose size they can vary. Again three rules are imposed, but now in a way that the roles of monsters and globes are interchanged, i.e., monsters correspond to discs (again in inverted order of size) and globes to pegs. The experiments revealed an extreme increase in difficulty from the ToH via the MaG move problem to the MaG change problem, despite the fact that all state graphs are isomorphic to $H_3^3$ and the tasks administered corresponded to the same $1/3/2 \to 3/2/1$, which requires 5 steps. Although horizontal transfer effects had been observed for problem isomorphs of similar outer appearence (cf. [21]), it would be interesting to know if there is *negative* vertical transfer, e.g., whether subjects well-trained with the ToH perform worse in MaG change tasks where static and dynamic components of the puzzle have switched their roles. Among the various representations, e.g., of the ToH in [33], one might actually include the most basic problem isomorph, namely to ask subjects to draw a shortest path between two given dots on the *abstract problem space*, i.e., the graph of Fig. 3 without labels. In this form, the ToH has been tested with animals, who found the shortest path in a maze formed after $H_3^3$ (see [25])!

A distinctive feature of the graphs $H_3^3$ and $L_{123}^3$ is their *maximal degree*. The *degree* of a vertex is the number of direct neighbors, i.e., the number of edges to which the vertex belongs. It represents the number of alternatives a subject has to make a move when in a certain state of the puzzle. The maximal degree of $H_3^3$ is $\Delta(H_3^3) = 3$, while $\Delta(L_{123}^3) = 4$. (Numerical values for other tower puzzles can be found in [13, Tables 1 and 2].) This shows that the task to find a walk, i.e., a sequence of moves, from an initial to a prescribed goal state, is facilitated in the ToL, but the task to find a *shortest* path is more difficult here because of the larger number of alternative paths available. On the other hand, a higher number of *optimal* solutions for a specific task will lead to a better performance as has been demonstrated in [30] by imbedding tasks from $L_{123}^3$ into $L_{333}^3$, i.e., extending the space on the pegs. A comparison of tasks with equal length but different numbers of optimal solutions, as performed in [24], supports the presumption that the difficulty of a task can not just be deduced from the number of steps necessary to solve it.

### *4.1. Connectedness*

In view of the psychological distinction between well- and ill-structured problems (cf. [8]), the most important property of both $H_3^3$ and $L_{123}^3$ is that they are *connected*, that is, every task can be solved: given any two vertices, there is always at least one (shortest) path leading from one to the other. This property extends (by mathematical induction) to Hanoi graphs $H_3^n$ of higher exponent, i.e., with an arbitrary number $n$ of discs. In fact, they are hamiltonian; cf. [14, Theorem 1]. The London graph $L_{123}^3$ is not hamiltonian; however, there are paths containing every vertex, but whose end-vertices are not linked by an edge.

The concept of connectedness can also be employed to find out which generalizations of Shallice's ToL (cf. [27]) are meaningful for psychological tests. In [29], Tunstall proposed a ToL with four balls and all three pegs extended to hold one more ball each, in our notation $L^4_{234}$. The advantage of this variant is a larger choice of more challenging tasks. But is the graph, already rather complicated to draw (e.g., it is not *planar* anymore, i.e., edges must cross outside vertices), still connected and therefore every task solvable? Here mathematical analysis comes in handily.

**Theorem** [9, Lemma 18] The graph $L^n_{h_1\ldots h_p}$ is connected, if and only if $n \leqslant \sum_{k=1}^{p-1} h_k$, i.e., if it is possible to distribute the balls in such a way that the tallest peg remains empty.

**Proof.** The theorem is an interesting instance of an obviously necessary condition being (non-obviously) sufficient as well. In fact, with $1 + \sum_{k=1}^{p-1} h_k$ balls present, the ball on bottom of (the tallest) peg $p$ can not be moved anymore. So we have to show that we can handle $\sum_{k=1}^{p-1} h_k$ balls, because the case of fewer balls can be reduced to that by introducing virtual balls, which can afterwards be ignored again; cf. (a) in Fig. 5, where we illustrate the formal proof with the example to get from state $1/2/43$ to $/4/231$ in Tunstall's ToL $L^4_{234}$, which evidently fulfills the condition of the theorem.

We will now describe a procedure that yields a (not necessarily optimal) solution for any arbitrary task. This can be reduced to proving the following: for any state with pegs 1 to $p-1$ completely filled and peg $p$ empty it is possible to interchange two balls $a$ and $b$ without changing the positions of the other balls. This is sufficient, because balls to be placed on peg $p$ can be arranged on top of the other pegs in an appropriate order ((b) in Fig. 5), and any such distribution of balls can be reached by repeated switches of two balls ((c) in Fig. 5). We may further assume that one of the two balls, say $a$, is the top ball on peg 1, because three such switches will cover the general case ((d) in Fig. 5). Now there are two possibilities.

If ball $b$ lies on peg 1 as well ((e) in Fig. 5), we move the top ball $c$ on peg 2 to peg $p$, then $a$ to peg 2 and all other balls above $b$ one by one to peg $p$, where there is still at least one space free to receive $b$. Its place is then filled by $a$, $b$ is moved to peg 2, all balls from peg $p$ except $c$ return to peg 1, then $b$ as well and finally $c$ to its original position. (In the example of Fig. 5, we can save one move of ball $b = 4$. That three moves of ball $b$ might be necessary in this step of the algorithm is demonstrated in Fig. 6 for the task "adb/cef/ to bda/cef/ " in $L^6_{333}$.)

If $b$ is originally not on peg 1 ((f) in Fig. 5), then we move all balls above it on the same peg to peg $p$, then $b$ on top of that, $a$ to the position $b$ just left, $b$ to the top of peg 1 and all balls from peg $p$ back to where they came from. □

Note that although the algorithm described in the proof of the theorem is constructive in the sense that it gives a solution to every task, this solution will, in general, not be of minimal length (which would be 7 in the example of Fig. 5).

As a consequence of the theorem we see that not only all tasks in Tunstall's variant $L^4_{234}$ of the ToL can be solved, but that it is even possible to add a fifth ball to arrive at $L^5_{234}$. Note that the maximal degree goes up to 6, which considerably increases the number of move alternatives for the problem solver. From the mathematical point of view it is therefore highly recommended to employ one of these two versions in tests of higher cognitive skills.

### *4.2. Distance*

With connectedness of a graph comes a *distance function* $\mathrm{d}$ which assigns to every pair $\{x, y\}$ of vertices the length $\mathrm{d}(x, y)$ of a shortest path from $x$ to $y$ (and vice versa). This notion allows for quantitative comparison of tower tasks. For instance, for a long time it had been assumed, and is still postulated in some studies, that the largest disc moves at most once in a shortest solution of a ToH task, just because it is the most obstructed of the discs given the divine rule. This is the basis for the "Sophisticated Perceptual Strategy" of H.A. Simon in [28, p. 272], who proposed it for tower-ending tasks, where it actually leads to the optimal solution (cf. [11, Lemma 1(i)]). It is also true that the largest disc cannot move more than twice in an optimal path between two arbitrary states, because otherwise it would return to a peg it already had left before, such that all its moves in between could be deleted; cf. [11, Lemma 1(ii)]. However, a quick look at $H^3_3$ in Fig. 3 shows that the one-move assumption is wrong: to get from $12/3/$ to $3/12/$, say, it is obvious that one needs 7 moves if disc 3 moves only once (in the middle of the path), but that 5 moves are sufficient if the first and last move is performed with disc 3. There are also tasks with two different optimal solutions depending on whether the largest disc moves once or twice, as, e.g., from $12/3/$ to $13/2/$. There seems to be no systematic experimental study to investigate preference for one of these optimal paths; for corresponding tests with $L^5_{555}$, see [31, p. 67ff]. A thorough mathematical analysis (cf. [12]) reveals that shortest paths with two moves of the largest disc are rare, so moving it only once is a good, albeit not optimal strategy for the ToH.

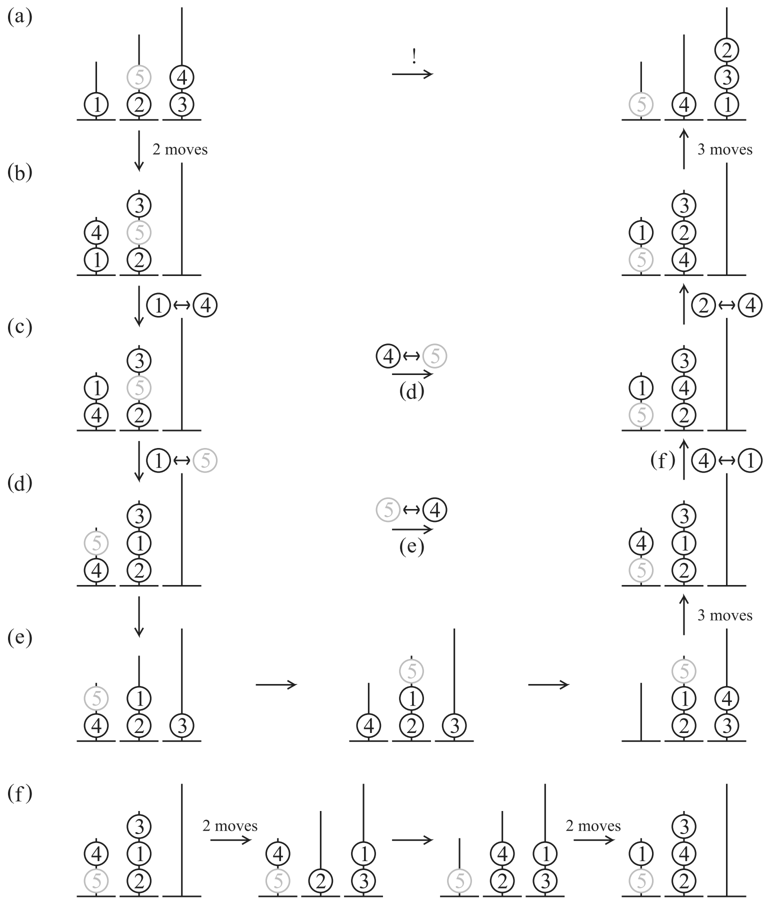

Fig. 5. Example for an application of the algorithm in the proof of the theorem.

This in turn can be transferred into a possible strategy for solving ToL tasks. We already observed that the graphs $H_3^3$ and $L_{123}^3$ are not isomorphic. However, they have certain features in common, for instance being composed of three substructures. In $H_3^3$ they are characterized by the three possible positions of the largest disc 3. For $L_{123}^3$ that role is taken by the color of the ball on bottom of the tallest peg. Again the corresponding strategy, namely to move that ball directly to its goal position, is good but not always optimal. This is demonstrated by the example to get from $/21/3$ to $2/3/1$, where for a minimal solution (of length 7) it is necessary to move the ball with color 2 to the tallest peg *before* ball colored 1.

Although the optimal path in the previous example is unique, there may be as many as eight different minimal solutions for other ToL tasks like, e.g., from $1/2/3$ to $1/3/2$. As mentioned before, this is caused by the higher maximal degree of $L_{123}^3$ as compared to $H_3^3$ and in fact for all graphs $H_3^n$ with arbitrarily many discs, there are at most two shortest paths for any task (cf. [11, Theorem 4]). Tasks involving a *perfect state*, i.e., with all discs on one and the same peg, have unique shortest solutions with disc $n$ moving at most once as indicated before.

A thorough analysis of distances on $H_3^n$ led to the impressive formula (cf. [11, Proposition 7])

$$\frac{466}{885}\,18^n - \frac{1}{3}\,9^n + \frac{6}{59}\left(2 + \frac{3}{17}\sqrt{17}\right)\cdot \left(\frac{1}{2}\left(5+\sqrt{17}\right)\right)^n - \frac{3}{5}\,3^n + \frac{6}{59}\cdot \left(2 - \frac{3}{17}\sqrt{17}\right)\left(\frac{1}{2}\left(5-\sqrt{17}\right)\right)^n$$

for the sum of the lengths of all tasks, from which the average distance of two states can be calculated as 4.043 and 8.204 for $H_3^3$ and $H_3^4$, respectively; the corresponding value for the classical ToL $L_{123}^3$ is 4.357, and for Tunstall's ToL $L_{234}^4$ it is 5.850. For other tower puzzles see [13, Tables 1 and 2].

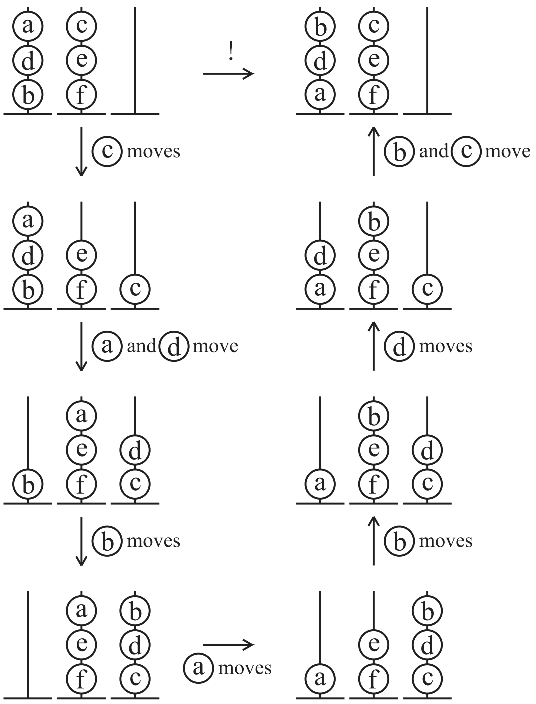

Fig. 6. Example for interchanging two balls on one peg in the proof of the theorem.

The maximal distance on a graph is called its *diameter* and represents the worst case, i.e., the longest among all shortest paths between vertices of the graph. The diameter is $2^n - 1$ for $H_3^n$, 8 for $L_{123}^3$, and 10 for $L_{234}^4$; again other values can be found in [13, Tables 1 and 2]. It turns out that the average distance is always a little larger than one half of the diameter. This is an indication that there are not too many tasks whose optimal solutions are parts of optimal solutions of other tasks (sometimes called "nested problems"). They would cause some boredom, but could nevertheless be incorporated into a sequence of tasks to find out about learning.

In the design of test series, another parameter is of some interest, namely the *eccentricity* $\varepsilon(v)$ of a vertex $v$, which is defined as the maximal distance to any other vertex. (The diameter is therefore the maximal eccentricity over all vertices.) In his original set of twelve tasks for the ToL (cf. [20, Appendix]), Shallice always used the same initial state, namely (an equivalent of) $/1/23$, whose eccentricity is 7 and therefore gives rise to sufficiently many tasks with different lengths of shortest solutions. Moreover, Shallice's start vertex is of maximal degree 4, which makes the choice for the first move rather difficult for subjects. The issue of degree, or *move choices* as it has been called in [2, p. 475], has not been given sufficient attention in psychological tests with the ToL, where *state hierarchy* was emphasized, i.e., whether a state is tower-like, partially tower-like or flat (cf. the overview on structural problem parameters in [17, p. 3]). While this may have an impact on planning for the goal state (cf. infra), the most important factor for a subject in an intermediate state and even more so in the initial state, where there is no "step back", is the number of choices for an action, i.e., move. In [2] only the average degree, which is 3 in the *degree balanced* graph $L_{123}^3$ with 12 vertices for each of the degrees 2, 3, and 4, respectively, has been accounted for, whereas start and goal hierarchy have been considered individually for each task. State hierarchy and degree are *not* equivalent though, as can be seen from Fig. 4.

Yet another measure, namely the average eccentricity of the graph, can be useful in the decision for a variant of classical tower puzzles. It has been derived for Hanoi graphs $H_3^n$ in [15, Proposition 4.4] as $\frac{14}{15} \cdot 2^n - \frac{2}{3} - \frac{3}{5} \cdot \left(\frac{1}{3}\right)^n$ and yields information whether randomly chosen states will be suitable for starting positions of a list of tasks. Similarly, based on average distances, tasks can be chosen randomly in order to produce test series unbiased by the choice of the experimenter.

To summarize the mathematical implications state graphs engender on the problem structure and selection of tasks, we emphasize that the degree of a vertex corresponds to the move choices and that therefore a large maximal degree may cause difficult situations for the planning subject. The eccentricity of the start vertex (or average eccentricity if the latter is chosen randomly) helps the experimenter in selecting in order to obtain a sufficiently challenging set of goal states. Global parameters of the graph like connectedness and planarity guarantee feasibility of all tasks and a clear graphical representation of the solution paths, respectively. Hamiltonicity opens a way to design new types of tasks, namely to find long, non-self-crossing tours (cf. [10]). The distance of two vertices in the state graph is, of course, one of the main measures of difficulty of individual tasks, and again the average distance may serve to estimate the mean level of difficulty (in this restricted sense) if tasks are chosen randomly in an experiment. Finally, the diameter of the graph provides the upper bound for the length of a solution of an arbitrary task.

But there is at least one more lesson to be learned from the state graphs of tower puzzles, namely from their symmetries.

## 5. Symmetries of tower graphs

Useful information about tower tasks and for the design of tests based on them can also be drawn from symmetry properties of the state graphs. Since every move can be performed in both directions, these graphs are undirected and therefore there is a symmetry in switching initial and goal state of any task. (There are, however, some variants where certain types of moves are disallowed, like in the *cyclic ToH*, where the three pegs are arranged in a circle and one can move a disc only clockwise between the pegs. We will not address these versions here.) The corresponding tasks seem not to be equivalent from a psychological point of view though. This may not be too pronounced in the classical ToL, but the task to get from a perfect tower to a given "flat" distribution of discs in the ToH seems to be harder than vice versa. (Contrary to everyday experience, this means that it is demanding to get from order to disorder. The reason, however, is that here the disorder is prescribed in a deterministic way!) A sequence of tasks to be performed in a tower test should therefore contain a task and its inverse mixed in; see the experiment on $L^5_{555}$ in [31, p. 64–69]. This can lead to some insight into the way strategies are employed in the solution process. The diagnostic value of considering goal hierarchy is demonstrated in [22].

The graph $H^3_3$ in Fig. 3 has an obvious 6-fold symmetry: rotations by $120^\circ$ around the center and reflections with respect to axes through perfect states and perpendicular to the opposite base of the triangular graph. It can be shown that these are in fact the only *automorphisms* of $H^3_3$, i.e., graph isomorphisms preserving the vertex set, namely the set of states of the ToH with three pegs and three discs. They can be interpreted as permutations of the pegs. Therefore, the $3^n(3^n - 1)$ non-trivial tasks for $H^n_3$ come in blocks of six (mathematically) equivalent tasks with the pegs permuted in the initial and goal states. Two such tasks, like, e.g., $3/1/2$ to $/2/13$ and $2/3/1$ to $13//2$, have been called "iso-problems" in psychology literature (cf. [1]), and it is very useful to include such a pair of tasks in a series forming a test to find out about learning and memory processes.

Such tests have been performed (cf. [6]) with the analogous iso-problems of ToL tasks. Here the symmetries come from permutations of the ball colors. This again corresponds to $120^\circ$ rotations of the graph $L^3_{123}$ of Fig. 4, but instead of direct reflections, we have to turn the graph inside out and then reflect. Again the $1260$ non-trivial tasks fall into $210$ classes of iso-problems. This relatively small number of non-equivalent tasks sheds some doubt on the value of the classical ToL for ample psychological test series. The ToH with $4$ discs already has $1080$ non-equivalent tasks to offer, Tunstall's ToL has $2893$. For other ToL versions these considerations become more involved because symmetries can overlap and deeper mathematical tools (Burnside's lemma) have to be employed (cf. [9]). For instance, the graph $L^3_{333}$ underlying experiments in [16,18] with a repeated presentation of equivalent tasks avoiding the awareness of subjects of these repetitions, has 95 sets of 36 iso-problems each, 6 sets of 18, and 1 set of 12. More numerical results can be found in [13, Table 3].

## 6. Computerized test tool

The tests in [6] have been conducted with the aid of a computerized test tool which is based on the mathematical theory presented in this note and described in detail in [13, Section 2]. Its main feature is the capability to plan, design and perform a variety of well-defined experiments with the ToH and the ToL on a tablet PC, which makes it suitable also for clinical use. One of the many options for evaluation of the subject's performance is to draw the walk (s)he has gone during the test on the graph of the respective puzzle. This picture

(cf. [13, Fig. 6]) also contains the optimal path(s) for comparison. For the lay-out of these graphs it is desirable (but not necessary) that they are planar. This is, however, not the case for most variants of the ToL, like, e.g., $L^3_{222}$ and consequently Tunstall's ToL, and virtually all tower graphs for puzzles with more than three pegs (cf. [14, Theorem 2]). In these cases, appealing alternative drawings are being developped.

## 7. Conclusion

The ToH and in more recent years the ToL have been employed in a plenitude of tests in the psychology of problem solving. However, the mathematical base of the underlying puzzles was not well understood and documented. In this note we summarized this theory to show how useful a better understanding can be for the design and the interpretation of those tests and to avoid mistakes like the "one-move-of-the-largest-disc" myth for the ToH. The fundamental notion of a graph, which also makes its appearance in other parts of neurosciences (cf., e.g. [4]), not only brings order into the numerous variations of tower transformation tasks (the "Five-disc Tower of London" of [31] is just $L^5_{555}$!), but provides also quantitative measures for their applicability and qualitative insights into the problem structure of the variants. If the ToL was originally invented because the classical ToH was believed to be less flexible and rich in offering a variety of tasks, it now turns out that $L^3_{123}$ is perhaps too simple for more distinctive goals of tower puzzle tests. It is definitely desirable to extend studies like [7], which was carried out with healthy participants, to ToH tasks involving 5 or even 6 discs, say. In any case, the Hanoi and London communities, largely segregated so far, should learn from each other and compare advantages and disadvantages of both puzzle types for their tests. And finally, the mathematical analysis can help in the development of new tests based on other ToL versions like $L^4_{234}$.

## Acknowledgements

Christoph P. Kaller (Freiburg) and two anonymous reviewers provided very constructive suggestions. I am also grateful to Ciril Petr (Maribor) for a valuable discussion and technical help. My travel to Heybeliada was supported by a mobility grant from the Bavarian Research Alliance, Munich. I thank Ayşe Erzan, Suat Mertoğlu, and Ekrem Tok for their hospitality in İstanbul.
